# Characterization of a novel cell wall hydrolase CwlE involved in *Bacillus thuringiensis* subsp*. israelensis* mother cell lysis

**DOI:** 10.3389/fmicb.2023.1250542

**Published:** 2023-09-27

**Authors:** Lixin Huang, Guangjie Han, Neil Crickmore, Chuanming Li, Yang Xia, Fuping Song, Jian Xu

**Affiliations:** ^1^Department of Applied Microbiology, Lixiahe District Institute of Agricultural Sciences in Jiangsu/National Agricultural Experimental Station for Agricultural Microbiology in Yangzhou, Yangzhou, China; ^2^Department of Biochemistry, School of Biological Sciences, University of Sussex, Brighton, United Kingdom; ^3^State Key Laboratory for Biology of Plant Diseases and Insect Pests, Institute of Plant Protection, Chinese Academy of Agricultural Sciences, Beijing, China

**Keywords:** *Bacillus thuringiensis* subsp. israelensis, mother cell lysis, cell wall hydrolase, transcriptional regulation, encapsulation

## Abstract

Cell wall hydrolases are ubiquitous among spore-form bacteria and essential for mother cell lysis. In this study, a novel cell wall hydrolase gene *cwlE* involved in mother cell lysis was characterized from *Bacillus thuringiensis* subsp*. israelensis* (Bti) strain Bt-59. *cwlE* was specifically expressed in Bti and located in the large plasmid carrying the insecticidal genes. The encoded CwlE protein consists of a Mur*N*Ac-LAA domain and two highly conserved catalytic residues (E26 and E151). The recombinant CwlE-His protein was able to digest the cell wall of Bti, indicating that CwlE is an *N*-acetylmuramoyl-_L_-alanine amidase. Transcriptional analysis indicated that *cwlE* began to express at the early stage of stationary phase and was controlled by SigE. Single mutation of *cwlE* gene delayed Bti mother cell lysis, while double mutation of *cwlE* and *sigK* completely blocked Bti mother cell lysis. After exposure to UV light to deactivate the crystal proteins, the level of decrease of insecticidal activity against mosquito larvae of Bt-59 (Δ*cwlE-sigK*) was less than that observed for Bt-59. This study elucidates the mechanism of Bti mother cell lysis and provides an effective strategy for mosquito control using Bt products with increased persistence.

## Introduction

Mosquitoes are tropical disease vectors for transmitting malaria, dengue, filariasis, and yellow fever, posing an enormous public health menace (Reisen, 2010; Zhang et al., 2017). Although chemical pesticides are the most widespread way to control mosquitoes, they are losing the advantage due to pest resistance and environmental pollution (Maciel-de-Freitas et al., 2014; Omotayo et al., 2022). *Bacillus thuringiensis* subsp. *israelensis* (Bti) is a gram-positive bacterium that forms a parasporal crystal composed of protein protoxins with specific insecticidal activity against dipteran larvae (Federici et al., 2013; Ben-Dov, 2014). Long-term exposure of mosquito larvae to Bti did not evolve into resistance to this microbial larvicide, which supports its sustainable use for integrated control practices (Carvalho et al., 2018). The low probability of development of resistance to Bti is attributed to the synergistic interaction among its four main Cry toxins, namely Cry4Aa, Cry4Ba, Cry11Aa, and Cyt1A (Vasquez et al., 2009). However, the main disadvantage of Bti is its short efficacy duration against mosquito larvae, limiting its widespread use as a larvicide.

Like other *B. thuringiensis* (Bt) strains, the durable spore and the parasporal crystal are set free with Bti mother cell lysis at the late sporulation stage (Ben-Dov, 2014). Then, the exposed insecticidal crystal proteins (ICPs) are easily inactivated by environmental factors, especially solar irradiation (Ramirez-Lepe et al., 2003; Zogo et al., 2019). An asporogenic Bt strain that expressed a chimeric *cry1C/Ab* gene showed high potency against various lepidopteran pests, and parasporal crystals produced by the strain remained encapsulated within the mother cells, which protected them from UV light degradation (Sanchis et al., 1999). Therefore, it is feasible to achieve the protection of ICPs from solar inactivation by knocking down the crucial cell wall hydrolase gene to block Bti mother cell lysis.

Three cell wall hydrolase genes (*cwlB*, *cwlC*, and *cwlH*) were identified in *Bacillus subtilis*, a well-established model for mother cell lysis (Vollmer et al., 2008). Except for the expression of *cwlB* in vegetative growth stage, *cwlC* and *cwlH* were expressed during the sporulation phase (Foster, 1992; Kuroda et al., 1993; Nugroho et al., 1999). Deleting of a single hydrolase gene did not affect mother cell lysis, but both *cwlB*-*cwlc* and *cwlB*-*cwlH* mutations blocked mother cell lysis of *B. subtilis* (Nugroho et al., 1999). While in *B. thuringiensis* subsp. *kurstaki* (Btk) strain HD73, the deletion of *cwlC* completely blocked Btk mother cell lysis, which lead to ICPs encapsulated in the mother cell (Chen et al., 2018). Based on the previous study, although CwlC of Bti showed 97% amino acid sequence identity with that of Btk, the deletion of *cwlC* just delayed the lysis of mother cells in Bti (Huang et al., 2022). The role of CwlC from Bti performed during the mother cell lysis was quite different from that of Btk, indicating that the hydrolysis of Bti mother cell wall involves a complex lysase system.

In the previous study, three cell wall hydrolase genes (*cwlB*, *cwlC*, and *cwlE*) were identified in the genome of Bti strain Bt-59 (Huang et al., 2022). *cwlB* and *cwlC* were regulated by SigK, a critical regulatory factor in the sporulation phase regulating genes involved in the formation of the outer wall of the spore, mother cell lysis, and spore germination (Zheng and Losick, 1990; Nugroho et al., 1999; Huang et al., 2022). The *sigK* and *cwlC* mutation strains of Bti were constructed in the previous study. As the key Sigma factor in regulating spore maturation and germination, the absence of *sigK* inhibits spore formation in Bti (Peng et al., 2016; Xu et al., 2020). Due to the major insecticidal genes of Bti are controlled by the sporulation-specific sigma factor SigE, the deletion of *sigK* did not have a significant impact on the production of ICPs in Bt-59 (Deng et al., 2014; Xu et al., 2020). In addition, the deletion of *sigK* or *cwlC* just delayed but not completely blocked Bti mother cell lysis (Xu et al., 2020; Huang et al., 2022). Moreover, *cwlE* was the only hydrolase gene with high expression level in the *sigK* mutant of Bti, indicating that *cwlE* was not controlled by SigK and CwlE was a critical autolysin leading to Bti mother cells lysis (Huang et al., 2022). Based on these studies, the double mutation of *cwlE* and *sigK* is likely to block Bti mother cell lysis without affecting its gene expression of ICPs.

This work analyzed the function and transcriptional regulation of *cwlE*, a new cell wall hydrolase gene of Bti. The *cwlE* mutation strain was constructed and the deletion of *cwlE* delayed Bti mother cell lysis. In addition, an ICPs encapsulation strain of Bti was constructed by deleting both *sigK* and *cwlE*. The result of insecticidal activity analysis showed that the *sigK*-*cwlE* mutant strain had better tolerance to ultraviolet than the Bti wild strain. Based on the characteristics analysis of CwlE and its role in mother cell lysis, we elucidated the mechanism of Bti mother cell lysis and constructed an ICPs encapsulation Bti strain with the improvement of insecticidal persistence.

## Materials and methods

### Bacterial strains, plasmids, and culture conditions

The strains and plasmids used in this study were showed in Table 1. *Escherichia coli* strains Top10, ET12567, and BL21 (DE3) were used for molecular cloning, extracting non-methylated vectors, and protein expression, respectively (Wang et al., 2006, 2022). Bti strain Bt-59 (CGMCC strain number 16821) and Btk strain HD73 (BGSC strain number BGSC 4D4) were used to clone the target gene, for transformations and monitor promoter activity. *E. coli* strains were cultured at 37°C in Luria-Bertani (LB) broth medium with 100 μg/mL ampicillin (Amp) if needed. Bt strains were cultured at 30°C in Schaeffer’s sporulation Medium (SSM, 8 g L^−1^ nutrient broth, 0.12 g L^−1^ MgSO_4_, 1 g L^−1^ KCl, 0.5 mmol L^−1^ NaOH) with 25 μg/mL erythromycin (Ery) if needed (Schaeffer et al., 1965).

**Table 1:** Strains and plasmids used in this study.

Strains or plasmids	Characteristic(s)^a^	Reference(s) or source
*E. coli* strains		
Top10	Cloning host	Huang et al. (2022)
BL21 (DE3)	Expression strain	Nugroho et al. (1999)
ET12567	For extracting non-methylated vectors	Wang et al. (2006)
BL21 (pET*cwlE*)	BL21 (DE3) with plasmid pET*cwlE*	This study
BL21 (pET*cwlE-*E26A)	BL21 (DE3) with plasmid pET*cwlE-*E26A	This study
BL21 (pET*cwlE-*E151A)	BL21 (DE3) with plasmid pET*cwlE-*E151A	This study
*Bacillus thuringiensis* subsp. *israelensis* strains		
Bt-59	Wild type strain with four main insecticidal protein genes (*cry4Aa*, *cry4Ba*, *cry11Aa*, and *cyt1Aa*) and three cell wall lyase genes (*cwlB*, *cwlC*, and *cwlE*)	Xu et al. (2020)
Bt-59 (Δ*cwlE*)	Bt-59 Δ*cwlE* mutant	This study
Bt-59 (Δ*sigK*)	Bt-59 Δ*sigK* mutant	Xu et al. (2020)
Bt-59 (Δ*cwlE-sigK*)	Bt-59 Δ*cwlE*-*sigK* mutant	This study
Bt-59 (Δ*cwlE*::*cwlE*)	Bt-59 (Δ*cwlE*) carrying plasmid pHTHF*cwlE*, Ery^r^	This study
Bt-59 (Δ*cwlE-sigK*::*cwlE*)	Bt-59 (Δ*cwlE-sigK*) carrying plasmid pHTHF*cwlE*, Ery^r^	This study
Bt-59 (P*_cwlE_*-lacZ)	Bt-59 carrying plasmid pHTP*_cwlE_*, Ery^r^	This study
*B. thuringiensis* HD73 strains		
HD73	Wild type strain with one insecticidal protein gene (*cry1Ac*) and two cell wall lyase genes (*cwlB* and *cwlC*)	Du et al. (2012)
HD (Δ*cwlC*)	HD73 Δ*cwlC* mutant, Kan^r^	Chen et al. (2018)
HD (Δ*cwlC*::*cwlE*)	HD (Δ*cwlC*) carrying plasmid pHTHF*cwlE*, Kan^r^, Ery^r^	This study
HD (P*_cwlE_*-lacZ)	HD73 carrying plasmid pHTP*_cwlE_*, Ery^r^	This study
HD (Δ*sigE*)	HD73 Δ*sigE* mutant, Kan^r^	Xu et al. (2020)
HD (Δ*sigE*) (P*_cwlE_*-lacZ)	HD (Δ*sigE*) carrying plasmid pHTP*_cwlE_*, Kan^r^, Ery^r^	This study
HD (*cwlE*)	HD73 carrying plasmid pHTHF*cwlE*, Ery^r^	This study
HD (Δ*sigE*) (*cwlE*)	HD (Δ*sigE*) carrying plasmid pHTHF*cwlE*, Kan^r^, Ery^r^	This study
Plasmid		
pMAD	Temperature-sensitive plasmid, 9.86 kb, Ery^r^	Erdonmez et al. (2014)
pHT315	*B. thuringiensis*-*E. coli* shuttle vector	Arantes and Lereclus (1991)
pMADΔ*cwlE*	pMAD containing *cwlC* deletion gene	This study
pHTHF*cwlE*	pHT315 containing the promoter, ORF, and the terminator sequences of *cwlE*	This study
pET28a	Expression vector, Amp^r^, 5.4 kb	Novagen
pET*cwlE*	pET28a containing *cwlE* gene, Amp^r^	This study
pET*cwlE*-E26A	pET28a containing *cwlE*^E26A^ gene, Amp^r^	This study
PET*cwlE-*E151A	pET28a containing *cwlE*^E151A^ gene, Amp^r^	This study
pHT304-18Z	Promoterless *lacZ* vector, Ery^r^, Amp^r^, 9.7 kb	Agaisse and Lereclus (1994)
pHTP*_cwlE_*	pHT304-18Z carrying P*_cwlE_*, Amp^r^, Ery^r^	This study

^a^Erm^r^ means erythromycin resistance, Kan^r^ means kanamycin resistance, and Amp^r^ means ampicillin resistance.

### DNA manipulation

Primers used in this study were synthesized in GENERAL BIOL (Hefei, China) and showed in Supplementary Table S1. PCR was performed using PrimeSTAR HS DNA polymerase (TaKaRa, Beijing, China) with corresponding primers. DNA fragments were purified with the TIANgel Midi Purification Kit (TIANGEN, Beijing, China) and plasmids were purified with the TIANprep Mini Plasmid Kit (TIANGEN, Beijing, China). The ClonExpress II One Step Cloning Kit (Vazyme, Nanjing, China) was used to clone the DNA fragments into the linearized vectors. Plasmids were introduced into the Bt cells by electroporation as previously described (Huang et al., 2022).

### Strain construction

The temperature-sensitive suicide plasmid pMAD with the *cwlE* mutation sequence was constructed to delete the *cwlE* gene in the Bt-59 genome (Supplementary Figure S1). A 908-bp fragment upstream of *cwlE* (fragment A) was amplified by PCR, and *cwlE-1* and *cwlE-2* were used as primers. A 1013-bp fragment downstream of *cwlE* (fragment B) was amplified by PCR, and *cwlE-3* and *cwlE-4* were used as primers. The *cwlE* mutation box was amplified using fragments A and B as templates and *cwlE-1* and *cwlE-4* as primers by overlapping PCR. The resulting fragment was cloned into the pMAD plasmid to generate pMADΔ*cwlE* plasmid. Then, the recombinant plasmid pMADΔ*cwlE* was transformed into Bt-59 and Bt-59 (Δ*sigK*) to generate the *cwlE* deletion mutant Bt-59 (Δ*cwlE*) and the *sigK* and *cwlE* double mutant Bt-59 (Δ*cwlE-sigK*).

A 2693-bp fragment containing the promoter, ORF, and terminator sequences of *cwlE* was amplified by PCR using HF*cwlE-1* and HF*cwlE-2* as primers and cloned into the pHT315 shuttle vector to generate pHTHF*cwlE* plasmid. Then, the recombinant plasmid pHTHF*cwlE* was transformed into Bt-59 (Δ*cwlE*), Bt-59 (Δ*cwlE-sigK*), and HD (Δ*cwlC*) to generate complemented strains Bt-59 (Δ*cwlE*::*cwlE*), Bt-59 (Δ*cwlE-sigK*::*cwlE*), and HD (Δ*cwlC*::*cwlE*), respectively.

To further verify the hydrolytic activity of CwlE, an 822-bp fragment of *cwlE* gene was amplified by PCR using pET-cwlE-F and pET-cwlE-R as primers and cloned into the pET28a plasmid. Site-directed mutagenesis of *cwlE* (E24A and E151A) was performed with the Fast Mutagenesis System kit (TransGen, Beijing, China). Then, the recombinant plasmid pET*cwlE*, pET*cwlE-*E24A, and pET*cwlE-*E151A were transformed into BL21 to generate strains BL21(pET*cwlC*), BL21(pET*cwlC-*E24A), and BL21(pET*cwlC-*E151A) for protein expression. Besides, the recombinant plasmid pHTHF*cwlE* was transformed into HD (Δ*cwlC*) generating complemented strain HD (Δ*cwlC*::*cwlE*) to analyze the degradation activity of CwlE on Btk cell wall.

A 767-bp promoter region upstream of the *cwlE* gene (P*_cwlE_*) was cloned into the linearized vector pHT304-18Z to construct a *cwlE* reporter vector. Then, the recombinant plasmids pHTP*_cwlE_* was transformed into cells of Bt-59, HD73, and HD (Δ*sigE*), generating strains Bt-59 (P*_cwlE_*-lacZ), HD (P*_cwlE_*-lacZ), and HD (Δ*sigE*) (P*_cwlE_*-lacZ), respectively. Besides, the recombinant plasmid pHTHF*cwlE* was transformed into HD73 and HD (Δ*sigE*) generating strains HD (*cwlE*) and HD (Δ*sigE*) (*cwlE*) to analyze the effect of *sigE* deletion on the expression level of *cwlE*.

### Expression and cell wall hydrolysis of recombinant CwlE protein

After strains BL21(pET*cwlE*), BL21(pET*cwlC-*E24A), and BL21(pET*cwlC-*E151A) were grown in LB liquid medium (Amp, 100 μg/mL) at 37°C for 4 h with shaking at 220 rpm, 0.5 mM isopropyl-beta-D-thiogalactoside (Sangon, Shanghai, China) was added. The bacteria were cultured at 16°C for another 14 h with shaking at 160 rpm to induce protein expression. Then, the recombinant CwlE proteins with a His tag were purified according to the method described previously (Zhang et al., 2020). After grown in LB liquid medium at 30°C for 6 h with shaking at 220 rpm, Bt-59 was collected by centrifugation at 10000 rpm. Then, the bacteria were resuspended with TK buffer (0.05 M Tris–HCl, 0.05 M KCl, pH 7.0) and disrupted with a FastPrep-24 (MPBiomedicals). Unbroken cells were removed by low-speed centrifugation (3,500 rpm, 5 min). The crude cell walls were pelleted at 13500 rpm for 5 min at 4°C. Finally, the pellets were resuspended in TK buffer. The purified CwlE proteins were added to the prepared cell wall of Bt-59 for hydrolytic activity analysis (Huang et al., 2022). After incubated at 37°C, the OD_540_ of the mixtures were measured at the designated time point (Nugroho et al., 1999).

### Analysis of the transcription start site

Total RNA was extracted from Bt-59 cultured in SSM at *T_15_* with TRIzol reagent (Invitrogen, USA). The transcription start site of the *cwlE* gene was determined with 5’ RACE according to the manufacturer’s instruction of HiScript-TS 5′/3’ RACE Kit (Vazyme, Nanjing, China). The primers used for 5’ RACE are listed in Supplementary Table S1.

### β-Galactosidase assays

Bt strains HD (P*_cwlE_*-lacZ) and HD (Δ*sigE*) (P*_cwlE_*-lacZ) were cultured in SSM (Ery, 25 μg/mL) at 30°C with shaking at 220 rpm. Two-milliliter bacterial suspensions of HD (P*_cwlE_*-lacZ) and HD (Δ*sigE*) (P*_cwlE_*-lacZ) were collected from *T_0_* to *T_20_* at 1-h intervals (*T_0_* is the end of the exponential phase and *T_n_* is n hours after the end of the exponential phase). The cells were harvested and the samples were prepared as previously described (Chen et al., 2018). Then, the specific β-galactosidase activity was measured and expressed as Miller units per milligram of protein (Chen et al., 2018). The values are the means for three independent experiments.

### Quantitative real-time PCR (qPCR)

Total RNA was extracted from Bt strains cultured in SSM at *T_0_*, *T_8_* and *T_16_* with TRIzol reagent (Invitrogen, USA). *T_0_* is the end of the exponential phase and *T_n_* is n hours after the end of the exponential phase. The purified RNA was reverse transcribed into cDNA using HiScript IV RT SuperMix for qPCR (gDNA wiper) (Vazyme, Nanjing, China). *RpsU* and *gatB* were chosen as reference genes (Reiter et al., 2011). qPCR was performed using specific primers (Supplementary Table S1) and ChamQ Universal SYBR qPCR Master Mix (Vazyme, Nanjing, China) with a 7,500 Fluorescence Quantitative PCR Instrument (ABI, California, USA). The specificity and qualified amplification efficiency of the specific primers were verified. Three biological replicates were designed. The relative expression level of each target gene was calculated according to the 2^-ΔCT^ method (Huggett et al., 2013).

### Assays of vegetative growth and Cry proteins production

The wild-type strain Bt-59, *cwlE* deletion mutant Bt-59 (Δ*cwlE*), and double mutant Bt-59 (Δ*cwlE-sigK*) were cultured in SSM medium at 30°C with shaking at 220 rpm. The growth curves of Bti strains were drew by measuring the OD_600_ values of the culture solutions every hour. After 12 h of cultivation, the number of colonies of Bt-59 and its mutants were calculated by the coating plate method. SDS-PAGE was used for the crystal protein production assay as described previously (Xu et al., 2020).

### Microscopic analysis

Bt strains were cultured in SSM medium at 30°C with shaking at 220 rpm to the certain time points. Samples were collected and centrifuged, and then sediments were suspended with the appropriate volume of deionized water. Bacterial suspension was placed on a glass slide, and then cell morphology was observed by optical microscopy (ZEISS, Germany).

### Bioassay of insecticidal activity

Bt-59 and Bt-59 (Δ*cwlE-sigK*) were cultured in a fermentation medium at 30°C with shaking at 220 rpm for 44 h as described previously (Huang et al., 2022). The fermentation media were serially diluted with water to the appropriate concentrations and used for the bioassay. Twenty larvae of *Culex pipiens* and *Aedes albopictus* in the early fourth instar were collected for bioassay in each treatment (Huang et al., 2022). Then the fermentation media were exposure to 365 nm UV light for 30 min used for the bioassay (Patel et al., 1996). The experiment was performed with three replicates at 28°C in a biochemical incubator for 24 h. The distilled water was used as a control.

### Sequence and data analysis

Vector NTI 11 and GENEDOC were used for sequence analysis. The primer design was performed by Primer Premier 5.0. The phylogentic tree was constructed with MEGA Microsoft Office Excel and GraphPad Prism 6 were used for data analysis. PoloPlus statistical software was used for estimating the LC_50_ values and 95% confidence intervals (CIs) (Straus, 2008). Lethal dose ratios (LC_50_ ratio, LCR) were calculated to compare natural variation between different treatments, and the LCR value was considered to be significant (*p* < 0.05) when the confidence intervals did not encompass the value 1 (Straus, 2008).

## Results

### Bioinformatic analysis of CwlE

**Figure 1:**
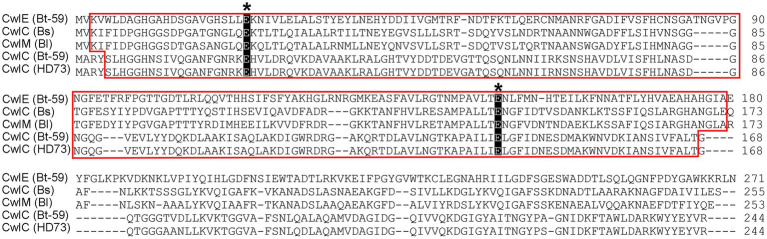
Bioinformatic analysis of cell wall hydrolases. Mur*N*Ac-LAA (red frame) represents the *N*-acetylmuramoyl-_L_-alanine amidase domain. The two conserved critical glutamic acid residues are marked with black asterisk. Bs and Bl are short for *Bacillus subtilis* and *B. licheniformis*, respectively. The NCBI accession number for CwlE (Bt-59), CwlC (Bt-59), CwlC (HD73), CwlC (Bs), and CwlM (Bl) are QCJ54933.1, QCJ49661.1, NC_020238.1, NP_389623.2, and P37134.1, respectively.

To identify the essential cell wall hydrolase in Bti mother cell lysis, a novel hydrolase gene named *cwlE* was identified from a 128 kb sized plasmid (CP039725.1) encoding multiple insecticidal protein genes of the strain Bt-59. *cwlE* is 816 base pairs in length and encodes a putative cell wall hydrolase with the Mur*N*Ac-LAA domain and two highly conserved catalytic residues E26 and E151 (Figure 1). The amino acid sequence identity showed CwlE shares 32% of its amino acid sequence identity with CwlM of *B. licheniformis*, 28% with CwlC of Bt-59, and 33% with CwlC of *B. subtilis*. The absence of a homologous sequence of *cwlE* in the genomes of bacteria other than Bti strains, as determined by the NCBI homology analysis, suggests that *cwlE* is a cell wall hydrolase gene uniquely expressed in Bti.

### Hydrolytic activity against cell wall of CwlE

**Figure 2:**
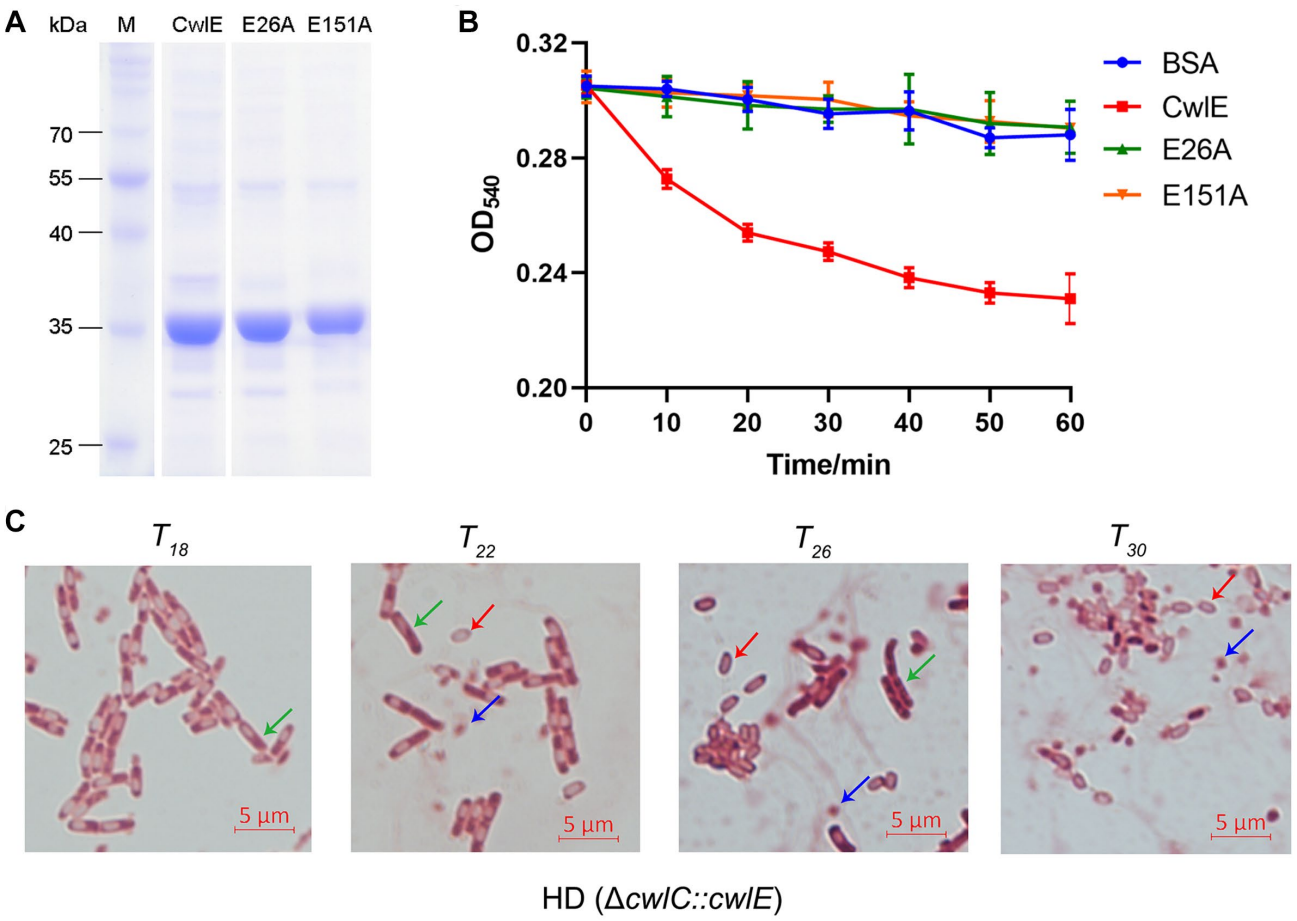
Expression of CwlE and its hydrolytic activity of cell wall. **(A)** SDS-PAGE analysis of recombinant CwlE and its mutant proteins purified by nickel column affinity chromatography. M, protein molecular size marker. CwlE, wild-type CwlE protein; E26A, replacement of glutamic acid with alanine of CwlE protein at position 24; E151A, replacement of glutamic acid with alanine of CwlE protein at position 151. **(B)** Digestion of *Bacillus thuringiensis* subsp*. israelensis* cell walls by purified CwlE and its mutant proteins. Cell walls incubated with addition of BSA protein were used as control. Each value represents the mean of at least three independent replicates. Error bars show standard deviations. **(C)** Mother cell lysis of HD (Δ*cwlC*::*cwlE*) was observed by optical microscopy after treatment with biological staining agent basic red at *T_18_*, *T_22_*, *T_26_*, and *T_30_*. *T_0_* is the end of the exponential phase and *T_n_* is n hours after the end of the exponential phase. Green arrows, red arrows, and blue arrows represent vegetative cells, spores, and crystal proteins, respectively. Scale bars, 5 μm.

To investigate the function of CwlE in Bti, the recombinant CwlE-His protein was purified using nickel column affinity chromatography. To determine whether the conserved glutamic acids are essential for enzymatic catalytic activity, two distinct single amino acid substitutions (E26A and E151A) were separately introduced into the CwlE-expressing strains using site-directed mutagenesis. SDS-PAGE showed that the molecular weight of the recombinant CwlE and its mutant proteins is approximately 35 kDa (Figure 2A). Then, mixtures of the recombinant proteins and the Bti cell wall were incubated at 37°C to determine the cell wall lytic ability of the CwlE and its mutant proteins. After incubation of CwlE with the cell wall for 60 min, the optical density (OD) of the mixture decreased by about 24% (Figure 2B). In contrast to the wild-type CwlE protein, the two mutant CwlE proteins (E26A and E151A) exhibited no catalytic activity against the Bti cell wall (Figure 2B).

After 15 days of growth in SSM medium the mother cells of Btk mutant strain HD (Δ*cwlC*) did not autolyze in a previous study (Chen et al., 2018). The *cwlE* gene containing its promoter region sequence was transferred into HD (Δ*cwlC*) strain to further determine the cell wall lytic ability of CwlE. Cells of HD (Δ*cwlC*::*cwlE*) began to autolyze at *T_22_* and completely autolyzed after 8 h of continuous culture (Figure 2C). The result indicated that the promoter of *cwlE* gene showed transcriptional activity in Btk, and CwlE could hydrolyze cell wall of HD73 and restore the phenotype of HD (Δ*cwlC*).

### Transcriptional regulation of the *cwlE* gene

**Figure 3:**
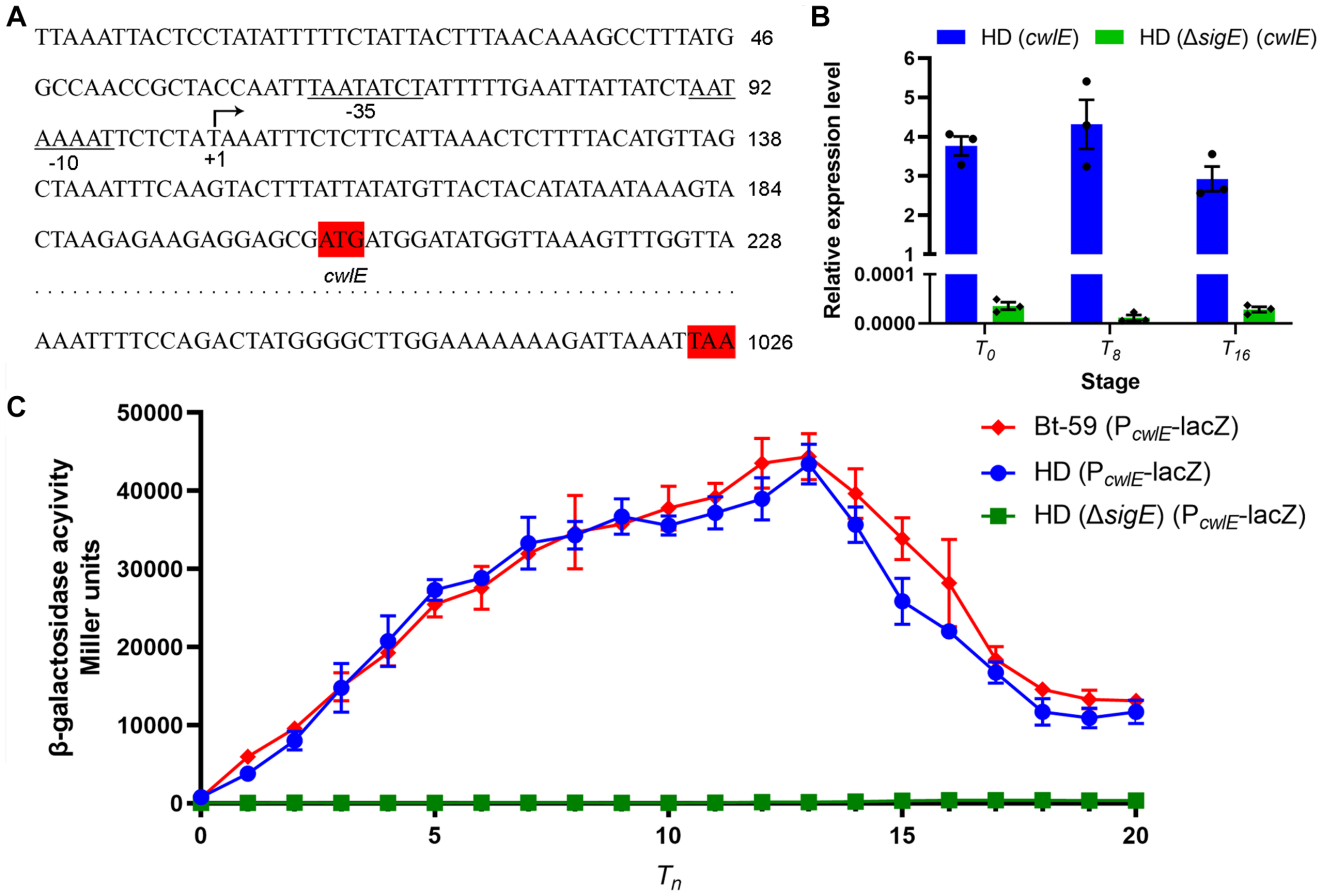
Analysis of promoter sequence and transcriptional regulation of the *cwlE* gene. **(A)** Sequence analysis of the promoter regions from Bt-59 *cwlE*. The predicted transcription start site T is denoted with arrow, while the putative −35 and − 10 motifs are underlined and denoted. The translation start codon and ending codon of *cwlE* are marked shaded in red. **(B)** Expression level of *cwlE* gene in HD (*cwlE*) and HD (Δ*sigE*) (*cwlE*) at *T_0_*, *T_8_*, and *T_16_*. Each value represents the mean of at least three independent replicates. Error bars represent the standard error of the mean. *T_0_* is the end of the exponential phase and *T_n_* is n hours after the end of the exponential phase. **(C)** Assays of β-galactosidase activity of the *cwlE* promoter in Bt-59, HD73, and HD (Δ*sigE*). *T_0_* is the end of the exponential phase and *T_n_* is n hours after the end of the exponential phase. Each value represents the mean of at least three independent replicates. The error bars represent standard deviations.

The result of 5’ RACE-PCR experiment showed that the transcription start site of *cwlE* gene is a T located 97 nucleotides upstream of its translational start codon ATG (Figure 3A). The putative *cwlE* promoter contains the sequences TAATATCT and AATAAAAT, which are located −35 and − 10 nucleotides upstream of the *cwlE* transcription start site, respectively (Figure 3A). These sequences are similar to those in the transcriptional regulatory factor SigE dependent promoters (KHATANHT and MATANNHT; H is A, C, or T; K is G or T; M is A or C; N is A, C, G, or T.) (Du et al., 2012), suggested that the *cwlE* promoter might be recognized and the expression of the *cwlE* gene was regulated by SigE.

The results of qPCR showed that the cwlE gene containing its promoter could be expressed in HD73, while it was almost undetectable in HD (Δ*sigE*) (Figure 3B). To further determine whether *cwlE* is regulated in a SigE-dependent manner, the Bt-59 (P*_cwlE_*-lacZ), HD (P*_cwlE_*-lacZ), and HD (Δ*sigE*) (P*_cwlE_*-lacZ) strains were constructed. β-Galactosidase activity assays showed that reporter vector was expressed both in Bt-59 and HD73, but not in *sigE* mutant strain of HD73 (Figure 3C). The result showed that the *cwlE* promoter could be recognized by SigE in Bti and Btk, and the expression of *cwlE* was controlled by SigE. In Bt-59, the expression of the reporter vector started at *T_0_*, reached a maximum at *T_13_*, and then decreased in the following time (Figure 3C). Furthermore, the transcriptional activity of the *cwlE* promoter did not differ significantly between Bt-59 and HD73 (Figure 3C).

### Effect of the *cwlE* and *cwlE*-*sigK* deletion on the vegetative growth and Cry gene expression

**Figure 4:**
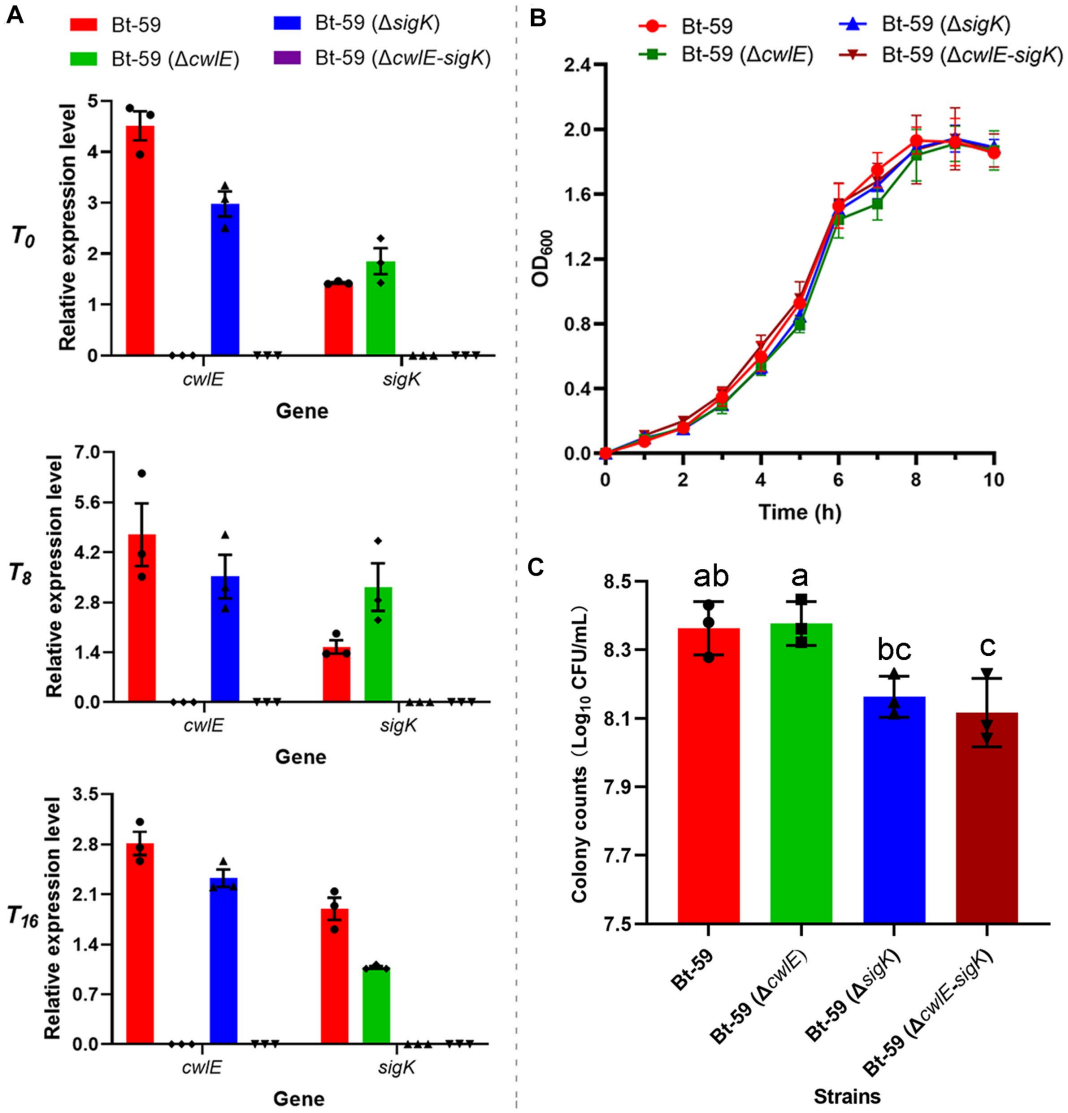
Effect of the *cwlE* and *cwlE-sigK* deletion on the vegetative growth and Cry proteins production of Bt-59. **(A)** The *cwlE* deletion mutants were verified by qPCR. Each value represents the mean of at least three independent replicates. Error bars represent the standard error of the mean. *T_0_* is the end of the exponential phase and *T_n_* is n hours after the end of the exponential phase. **(B)** Growth curves of Bt-59, Bt-59 (Δ*cwlE*), and Bt-59 (Δ*cwlE-sigK*). Each value represents the mean of at least three independent replicates. The error bars represent standard deviations. **(C)** After culturing in SSM medium for 10 h, the number of Bt-59, Bt-59 (Δ*cwlE*), and Bt-59 (Δ*cwlE-sigK*) cells was detected by viable count. Each value represents the mean of at least three independent replicates. The error bars represent standard deviations. Statistically significant difference analysis was performed by ordinary one-way ANOVA (*p* < 0.05).

Both the *cwlE* deletion mutant Bt-59 (Δ*cwlE*) and the *cwlE-sigK* double deletion mutant Bt-59 (Δ*cwlE-sigK*) have been constructed and verified (Figure 4A; Supplementary Figure S2). The growth curve of Bti strains during the nutritional period in SSM medium revealed that *cwlE* deletion or *cwlE* and *sigK* double deletion did not affect the nutritional growth of Bt-59 (Figure 4B). After being cultured in SSM medium for 12 h, the total number of cells of Bt-59, Bt-59 (Δ*cwlE*), Bt-59 (Δ*sigK*), and Bt-59 (Δ*cwlE-sigK*) were 2.3 × 10^8^ CFU/mL, 2.4 × 10^8^ CFU/mL, 1.5 × 10^8^ CFU/mL, and 1.3 × 10^8^ CFU/mL, respectively (Figure 4C). Unlike *cwlE* single mutation, *cwlE* and *sigK* double mutation affected the cells number of Bt-59, similar to *sigK* single mutation constructed previously (Xu et al., 2020).

**Figure 5:**
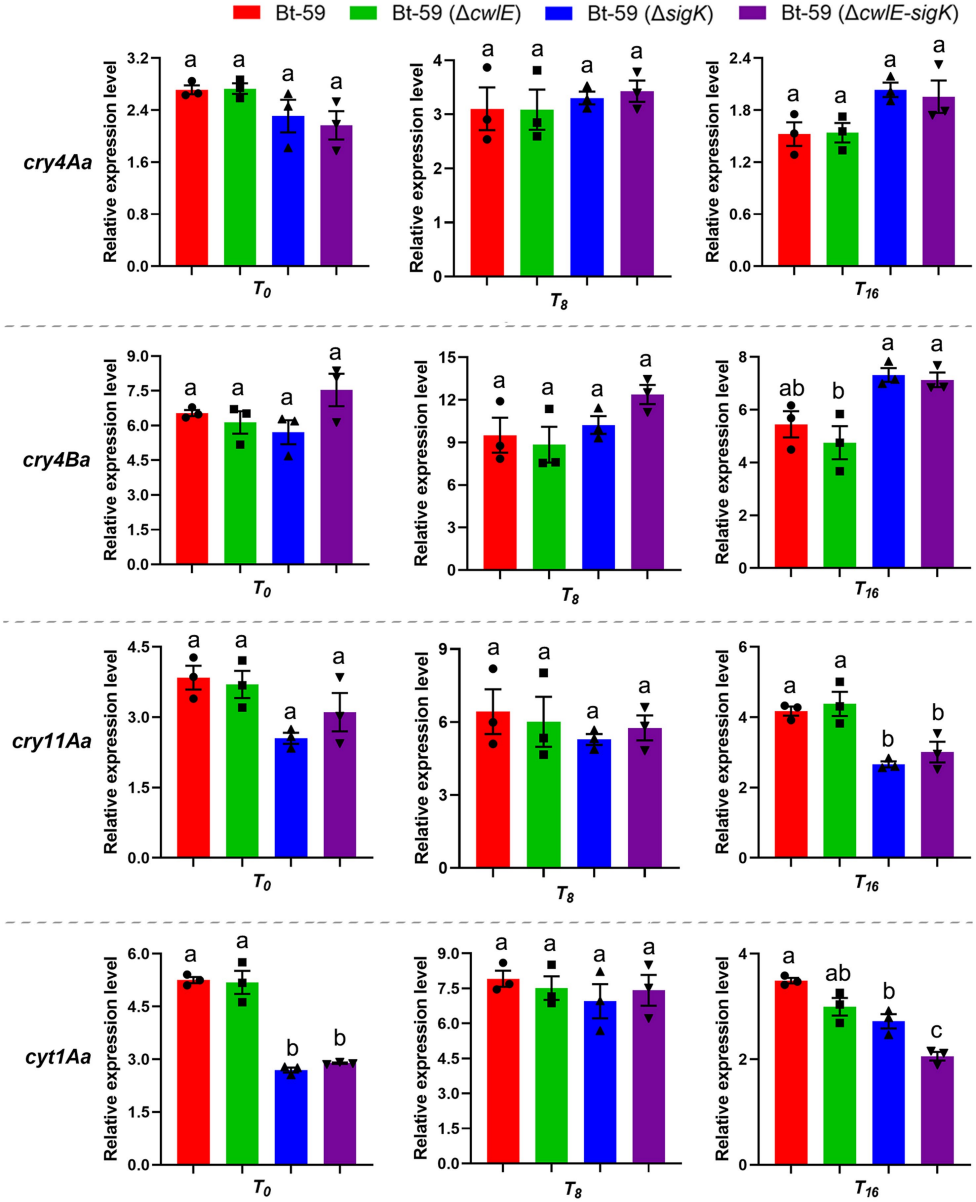
The expression levels of *cry4Aa*, *cry4Ba*, *cry11Aa*, and *cyt1Aa* in Bt-59, Bt-59 (Δ*cwlE*), Bt-59 (Δ*sigK*), and Bt-59 (Δ*cwlE-sigK*) at *T_0_*, *T_8_*, and *T_16_* were revealed by qPCR. Each value represents the mean of at least three independent replicates. Error bars represent the standard error of the mean. *T_0_* is the end of the exponential phase and *T_n_* is n hours after the end of the exponential phase. Statistically significant difference analysis was performed by ordinary one-way ANOVA (*p* < 0.05).

The expression levels of four main insecticidal protein genes (*cry4Aa*, *cry4Ba*, *cry11Aa*, and *cyt1Aa*) in Bt-59, Bt-59 (Δ*cwlE*), Bt-59 (Δ*sigK*), and Bt-59 (Δ*cwlE-sigK*) were revealed by qPCR (Figure 5). Compared with the wild strain, the deletion of *cwlE* or *sigK* did not affect the expression of *cry4Aa* and *cry4Ba* at *T_0_*, *T_8_*, and *T_16_. cry11Aa* showed similar expression levels in the four strains at *T_0_* and *T_8_*, while its expression was reduced in the mutants Bt-59 (Δ*sigK*) and Bt-59 (Δ*cwlE-sigK*) at *T_16_*. The deletion of *cwlE* did not affect the expression of *cyt1Aa*, while the expression of *cyt1Aa* was reduced in *cwlE* and *sigK* double mutation strain Bt-59 (Δ*cwlE-sigK*). Besides, Cry proteins production of Bt-59 and its mutants were examined by SDS-PAGE after being cultured in SSM medium for 24 h. The results showed that deletion of *cwlE* had no effect on Cry proteins production in Bti, while the *cwlE-sigK* deletion decreased the expression of Cry11Aa (Supplementary Figure S3).

### Deletion of the *cwlE* gene delayed mother cell lysis

**Figure 6:**
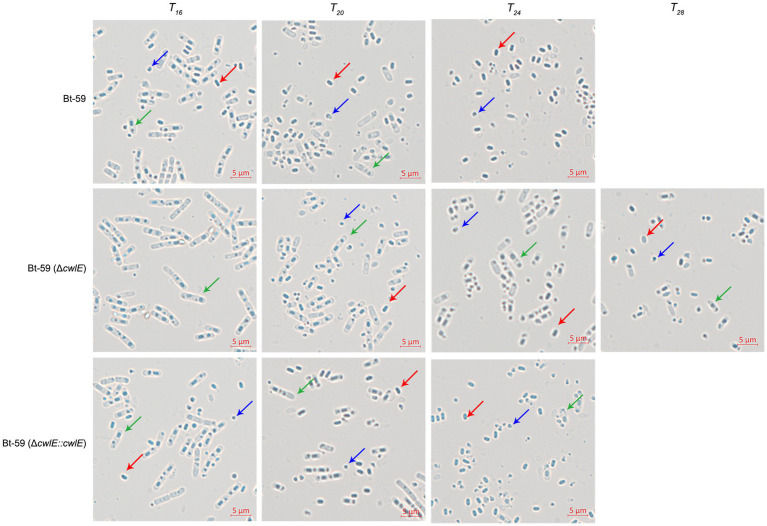
Effect of *cwlE* deletion on mother cell lysis observed by optical microscopy. Mother cell lysis of Bt-59 and Bt-59 (Δ*cwlE*::*cwlE*) was observed at *T_16_*, *T_20_*, and *T_24_*. Mother cell lysis of mutant strain Bt-59 (Δ*cwlE*) was observed at *T_16_*, *T_20_*, *T_24_* and *T_28_*. *T_0_* is the end of the exponential phase and *T_n_* is n hours after the end of the exponential phase. Green arrows, red arrows, and blue arrows represent vegetative cells, spores, and crystal proteins, respectively. Scale bars, 5 μm.

Optical microscopy was used to investigate the morphology of the Bt-59 and Bt-59 (Δ*cwlE*) grown in SSM medium to various growth phases (Figure 6). The result showed that cells of Bt-59 began to lyse at *T_16_*, while cells of Bt-59 (Δ*cwlE*) began to lyse at *T_20_*. Up to *T_24_*, the cells of Bt-59 were completely lysed, and the most of the cells of Bt-59 (Δ*cwlE*) were lysed. After another 4 hours of culture, the cells of Bt-59 (Δ*cwlE*) were completely lysed. In addition, the cells of *cwlE* gene restoration strain Bt-59 (Δ*cwlE*::*cwlE*) began to autolyze at *T_16_,* and virtually all of the cells were lysed at *T_24_*, which was similar to Bt-59.

### *cwlE* and *sigK* double mutant completely blocked mother cell lysis

**Figure 7:**
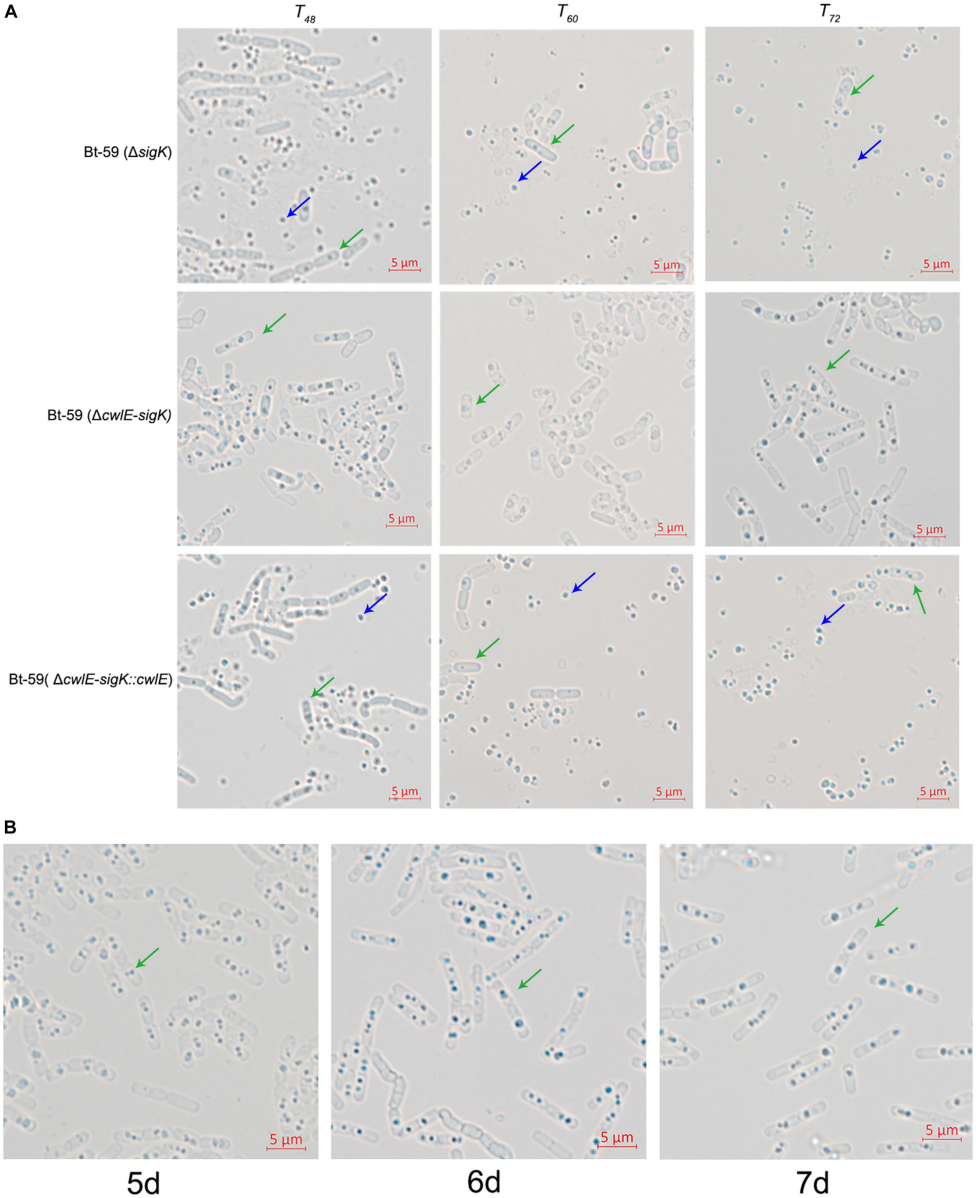
Effect of *cwlE* and *sigK* double deletion on mother cell lysis observed by optical microscopy. **(A)** Mother cell lysis of Bt-59 (Δ*sigK*), Bt-59 (Δ*cwlE*-*sigK*), and Bt-59 (Δ*cwlE*-*sigK*::*cwlE*) were observed at *T_48_*, *T_60_*, and *T_72_*. *T_0_* is the end of the exponential phase and *T_n_* is n hours after the end of the exponential phase. Green arrows and blue arrows represent vegetative cells and crystal proteins, respectively. Scale bars, 5 μm. **(B)** Mother cell lysis of mutant strain Bt-59 (Δ*cwlE*-*sigK*) was observed at 5 d, 6 d, and 7d. Green arrows represent vegetative cells. Scale bars, 5 μm.

To further verify the role of *cwlE* in mother cell lysis of Bti, the cell wall hydrolase gene *cwlE* and the transcription regulatory factor gene *sigK* double deletion mutant Bt-59 (Δ*cwlE-sigK*) was constructed based on the *sigK* mutant strain. Optical microscopy was used to examine the morphology of Bt-59 (Δ*sigK*) and Bt-59 (Δ*cwlE-sigK*) grown in SSM medium to various growth phases (Figure 7A). The result showed that parts of cells of Bt-59 (Δ*sigK*) were lysed at *T_48_*. Up to *T_72_*, the cells of Bt-59 (Δ*sigK*) were completely lysed, whereas mother cell lysis of Bt-59 (Δ*cwlE-sigK*) could not be observed. The cell morphology of the complemented strain Bt-59 (Δ*cwlE-sigK*::*cwlE*) was comparable to that of the *sigK* mutant strain. Furthermore, Bt-59 (Δ*cwlE-sigK*) was cultured in SSM for 7 days to further examine the mother cell lysis phenotype. The results showed that the cells of Bt-59 (Δ*cwlE-sigK*) still did not autolyze after 7 days of growth (Figure 7B).

### Insecticidal activity against mosquito larvae

**Figure 8:**
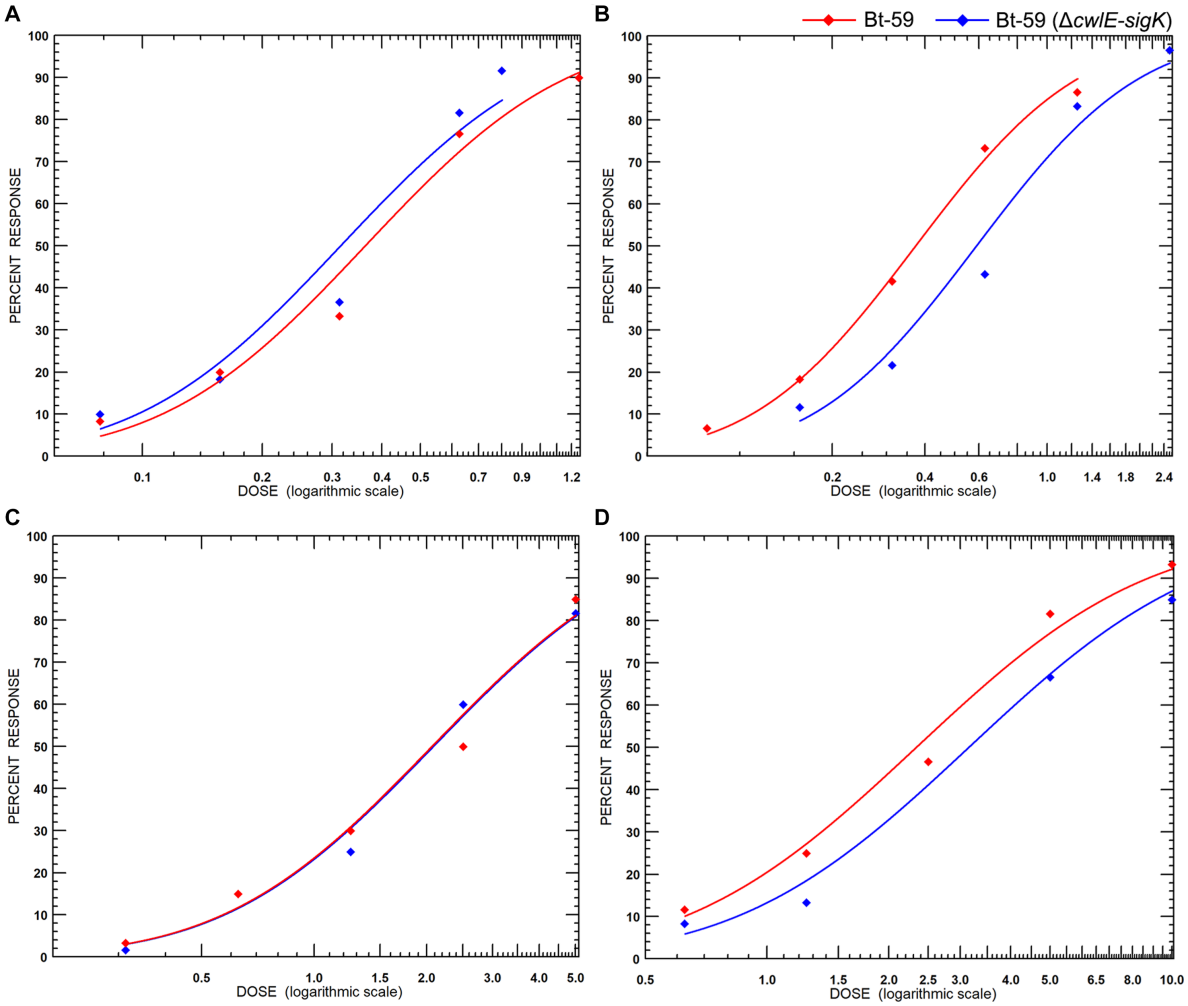
Insecticidal activity of Bt-59 and Bt-59 (Δ*cwlE*-*sigK*) against mosquito larvae. **(A)** Insecticidal activity of Bt-59 and Bt-59 (Δ*cwlE*-*sigK*) against *Culex pipiens*. **(B)** After exposure to UV light, the insecticidal activity of Bt-59 and Bt-59 (Δ*cwlE*-*sigK*) against *C. pipiens*. **(C)** Insecticidal activity of Bt-59 and Bt-59 (Δ*cwlE*-*sigK*) against *Aedes albopictus*. **(D)** After exposure to UV light, the insecticidal activity of Bt-59 and Bt-59 (Δ*cwlE*-*sigK*) against *A. albopictus*.

The early fourth-instar larvae of *Culex pipiens* and *Aedes albopictus* were fed with fermentation suspension serially diluted with water to the appropriate concentrations to determine the insecticidal activity of the *cwlE* and *sigK* double deletion mutant Bt-59 (Δ*cwlE*-*sigK*). The LC_50_ values of Bt-59 and Bt-59 (Δ*cwlE*-*sigK*) against *C. pipiens* were 0.314 μL/L and 0.364 μL/L, respectively (Table 2). After exposure to UV light, the insecticidal activity of Bt-59 and Bt-59 (Δ*cwlE*-*sigK*) against *C. pipiens* reduced by 86.62 and 2.47%, respectively (Table 2). The LC_50_ values of Bt-59 and Bt-59 (Δ*cwlE*-*sigK*) against *A. albopictus* were 2.066 μL/L and 2.075 μL/L, respectively (Table 3). After exposure to UV light, the insecticidal activity of Bt-59 and Bt-59 (Δ*cwlE*-*sigK*) against *A. albopictus* reduced by 52.76 and 12.43%, respectively (Table 3). The hypothesis of equality and parallelism showed that the *cwlE* and *sigK* double mutant did not affect the insecticidal activity of Bti against mosquito larvae (Figures 8A,C). In contrast, the level of decrease of insecticidal activity against mosquito larvae of Bt-59 (Δ*cwlE-sigK*) was less than that observed for Bt-59 after UV treatment (Figures 8B,D).

**Table 2:** Insecticidal activities of Bti strains against *Culex pipiens*.

Strain	Treatment	LC_50_ (95% CI) μL/L	Slope ± SE	χ^2^ (df)	LCR_50_ (95% CI)	Hypothesis of equality (χ^2^, *P*)	Hypothesis of parallelism (χ^2^, *P*)
Bt-59	-	0.314 (0.196–0.505)	2.712 ± 0.260	8.963 (3)	-	-	-
Bt-59 (Δ*cwlE*-*sigK*)	-	0.364 (0.260–0.523)	2.344 ± 0.230	4.343 (3)	0.862 (0.690–1.078)	Not Rejected (2.77, 0.251)	Not Rejected (1.13, 0.288)
Bt-59	UV	0.586 (0.409–0.841)	2.559 ± 0.244	5.200 (3)	-	-	-
Bt-59 (Δ*cwlE*-*sigK*)	UV	0.373 (0.315–0.445)	2.271 ± 0.227	0.719 (3)	1.570 (1.243–1.984)	Rejected (15.1, <0.05)	Not Rejected (0.75, 0.386)

**Table 3:** Insecticidal activities of Bti strains exposure to UV light against *Aedes albopictus*.

Strain	Treatment	LC_50_ (95% CI) μL/L	Slope ± SE	χ^2^ (df)	LCR_50_ (95% CI)	Hypothesis of equality (χ^2^, *P*)	Hypothesis of parallelism (χ^2^, *P*)
Bt-59	-	2.066 (1.748–2.487)	2.364 ± 0.245	2.127 (3)	-	-	-
Bt-59 (Δ*cwlE*-*sigK*)	-	2.075 (1.742–2.528)	2.229 ± 0.237	2.557 (3)	0.995 (0.771–1.285)	Not Rejected (0.16, 0.922)	Not Rejected (0.16, 0.692)
Bt-59	UV	3.156 (2.646–3.801)	2.147 ± 0.221	2.445 (3)	-	-	-
Bt-59 (Δ*cwlE*-*sigK*)	UV	2.333 (1.970–2.758)	2.332 ± 0.230	1.917 (3)	1.353 (1.057–1.732)	Rejected (6.24, <0.05)	Not Rejected (0.34, 0.563)

## Discussion

*N*-acetylmuramyl-_L_-alanine amidase contains the Mur*N*Ac-LAA domain and hydrolyzes the β-1,4 glycosidic bond between *N*-acetylglucosamine and *N-*acetylmuramic acid in polysaccharide chain, which is ubiquitous among spore-form bacteria and essential for the mother cell lysis (Vollmer et al., 2008; Bhagwat et al., 2020). CwlC is an essential cell wall hydrolase in Btk mother cell lysis and was distributed among the members of the *B. cereus* group (Chen et al., 2018). The CwlC exhibited the identical critical catalytic residues and conservative domains as Btk, whereas the deletion of *cwlC* delayed the lysis of mother cells in Bti (Huang et al., 2022). Although the mutation of *sigK* could inhibit the expression of the cell wall hydrolase genes *cwlC* and *cwlB*, it could not block cell wall lysis in Bti (Xu et al., 2020; Huang et al., 2022). This evidence demonstrates conclusively that Bti contains additional crucial cell wall hydrolases associated with mother cell lysis.

Bti’s novel cell wall hydrolase gene, *cwlE*, which is located on the large plasmid carrying the insecticidal genes, was characterized in this study. The absence of a *cwlE* homolog in other bacterial genomes, as determined by the NCBI homology analysis, indicates that *cwlE* is exclusively expressed in Bti. The CwlE is a new *N*-acetylmuramyl-_L_-alanine amidase, consisting of a Mur*N*Ac-LAA domain and two highly conserved catalytic residues (E26 and E151). Similar to the *cwlC* deletion mutant, the *cwlE* deletion mutant was unable to entirely prevent Bti mother cell lysis. In *B. subtillis*, cell wall lytic enzymes were functionally redundant because none of the single autolysin gene deletions affected mother cell lysis (Smith and Foster, 1995; Nugroho et al., 1999). However, the single mutant of autolysin genes delayed mother cell lysis in Bti, which differed from that in *B. subtillis*.

Sigma factors influence physiological process by regulating the transcription of various peptidoglycan hydrolase genes at various growth phases in Bacillus (Smith et al., 2000). Until now, the cell wall hydrolases involved in mother cell lysis were regulated by SigK in Bt strains (Yang et al., 2013; Chen et al., 2018; Huang et al., 2022). Analysis of promoter transcription activity showed that *cwlE* was regulated by SigE, which is the key Sigma factor regulating the expression of major insecticidal genes during the early sporulation stage in Bti (Deng et al., 2014). Our finding indicated that CwlE might be the key cell wall hydrolase causing the mother cell lysis in the *sigK* deletion mutant. To further verify the role of CwlE in Bti mother cell lysis, a double mutant strain was constructed by deleting of *cwlE* and *sigK* in this study. The results showed that deletion of *cwlE* and *sigK* could completely block Bti mother cell lysis, and cell wall lysis of double mutant could not be observed even after 7 days of culture in SSM medium. Therefore, we hypothesized that the cell wall lysis of Bti was accomplished through the synergy of the three hydrolases, CwlC, CwlB, and CwlE, and the absence of any cell wall hydrolases could delay mother cell lysis.

When Bt formulation is applied in the field, ultraviolet radiation is a key factor leading to crystal protein inactivation and reducing its insecticidal activity (Bravo et al., 2011). In the previous study, the mature crystal was produced and encapsulated in mother cells of a *sigK* deletion mutant of Bt strain, resulting in increased insecticidal activity against lepidoptera pests and greater resistance against UV radiation (Sanchis et al., 1999). In our study, the double mutant strain Bt-59 (Δ*cwlE*-*sigK*) showed increased insecticidal activity against the mosquito larva than the wild-type strain after exposure to UV light. By blocking mother cell lysis, it is possible to engineer a Bt strain with enhanced insecticidal persistence according to the current research.

We elucidated the function of CwlE in Bti mother cell lysis by deleting the hydrolase gene *cwlE* in this study. However, due to the lack of experimental data, we are not yet clear about the physiological function of *cwlE* expression at the early stage of stationary phase. Furthermore, compared to Btk, the reason why Bti requires more cell wall hydrolases to participate in mother cell lysis is also unclear. We speculate that due to the different midgut environments of different target insects, the synergistic effect of multiple cell wall hydrolases may allow Bti to release spores and crystal proteins faster in the mosquito midgut to complete population reproduction. Further investigations are needed to confirm this hypothesis.

## Conclusion

*cwlE* is a novel *N*-acetylmuramyl-_L_-alanine amidase gene in Bti strain Bt-59 and its expression is dependent on transcriptional regulation factor SigE. Deletion of *cwlE* delayed Bti mother cell lysis mother without impacting the vegetative growth and Cry proteins production. As a cell wall hydrolase uniquely expressed in Bti, CwlE is the key hydrolase causing the mother cell lysis in the *sigK* deletion mutant. The double mutation of *cwlE* and *sigK* completely blocked Bti mother cell lysis even after 7 days of growth. Due to the impact of the double mutation on the expression level of the major crystal protein genes were not significant, Bt-59 (Δ*cwlE*-*sigK*) still maintains high insecticidal activity against mosquito larvae. In addition, the level of decrease of insecticidal activity against mosquito larvae of Bt-59 (Δ*cwlE-sigK*) was less than that observed for Bt-59 after UV treatment. In summary, this study identified a novel and unique cell wall hydrolase gene *cwlE*, elucidated the mechanism of Bti mother cell lysis, and provided an effective strategy for mosquito control using crystal encapsulation of Bt strain with the increased resistance against UV light inactivation.

## Data availability statement

The original contributions presented in the study are included in the article/Supplementary material, further inquiries can be directed to the corresponding author.

## Ethics statement

The manuscript presents research on animals that do not require ethical approval for their study.

## Author contributions

JX conceived and designed research. LH, CL, and YX conducted experiments. NC and FS contributed new experimental idea. LH and GH analyzed data. LH wrote the manuscript. All authors read and approved the manuscript.
