# Supplementary material for: Characterization of a novel cell wall hydrolase CwlE involved in Bacillus thuringiensis subsp. israelensis mother cell lysis
Source: Front Microbiol. 2023 Sep 27;14:1250542. doi: 10.3389/fmicb.2023.1250542 (PMC10565116; doi:10.3389/fmicb.2023.1250542)
Supplement: Supplementary file 1 [file Data_Sheet_1.pdf]

Characterization of a novel cell wall hydrolase CwlE involved in *Bacillus thuringiensis* subsp. *israelensis* mother cell lysis

Lixin Huang[1], Guangjie Han[1], Neil Crickmore[2], Chuanming Li[1], Yang Xia[1], Fuping Song[3], Jian Xu[1*]

1. Department of Applied Microbiology, Lixiahe District Institute of Agricultural Sciences in Jiangsu/National Agricultural Experimental Station for Agricultural Microbiology in Yangzhou, Yangzhou 225007, China

2. Department of Biochemistry, School of Biological Sciences, University of Sussex, Falmer, Brighton BN1 9QG, United Kingdom

3. State Key Laboratory for Biology of Plant Diseases and Insect Pests, Institute of Plant Protection, Chinese Academy of Agricultural Sciences, Beijing 100193, China

*Corresponding Author:

Jian Xu

Department of Applied Microbiology, Lixiahe District Institute of Agricultural Sciences in Jiangsu/National Agricultural Experimental Station for Agricultural Microbiology in Yangzhou, Yangzhou 225007, China

Tel/ Fax: +86-0514-87637599

bio-xj@163.com

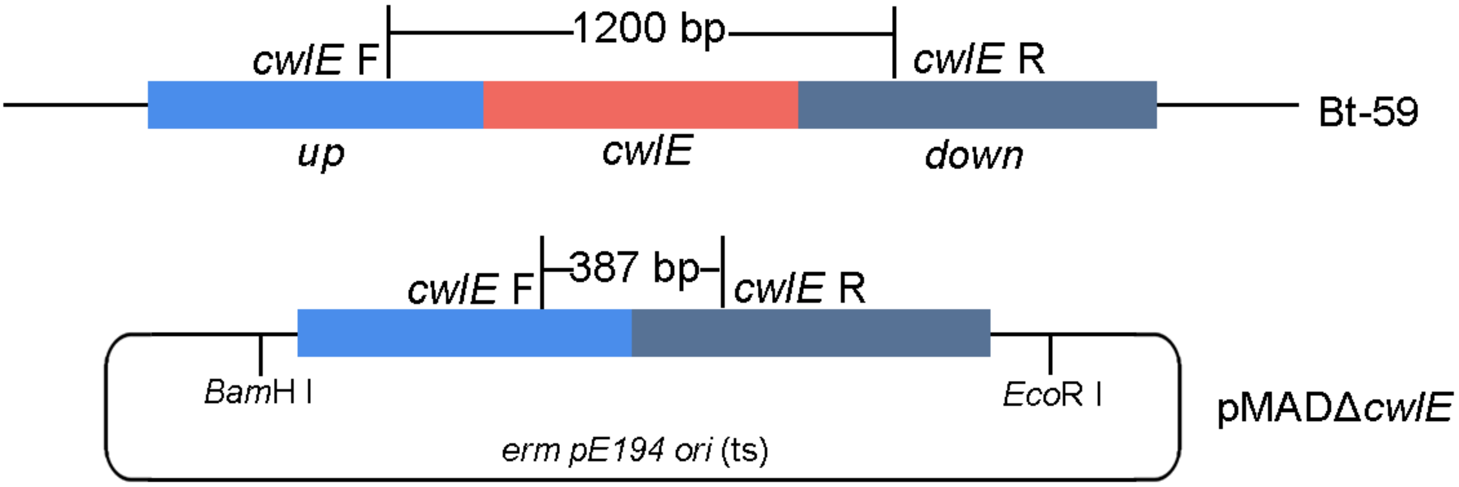

Figure S1. Schematic diagram of the *cwlE* deletion mutant strains construction.

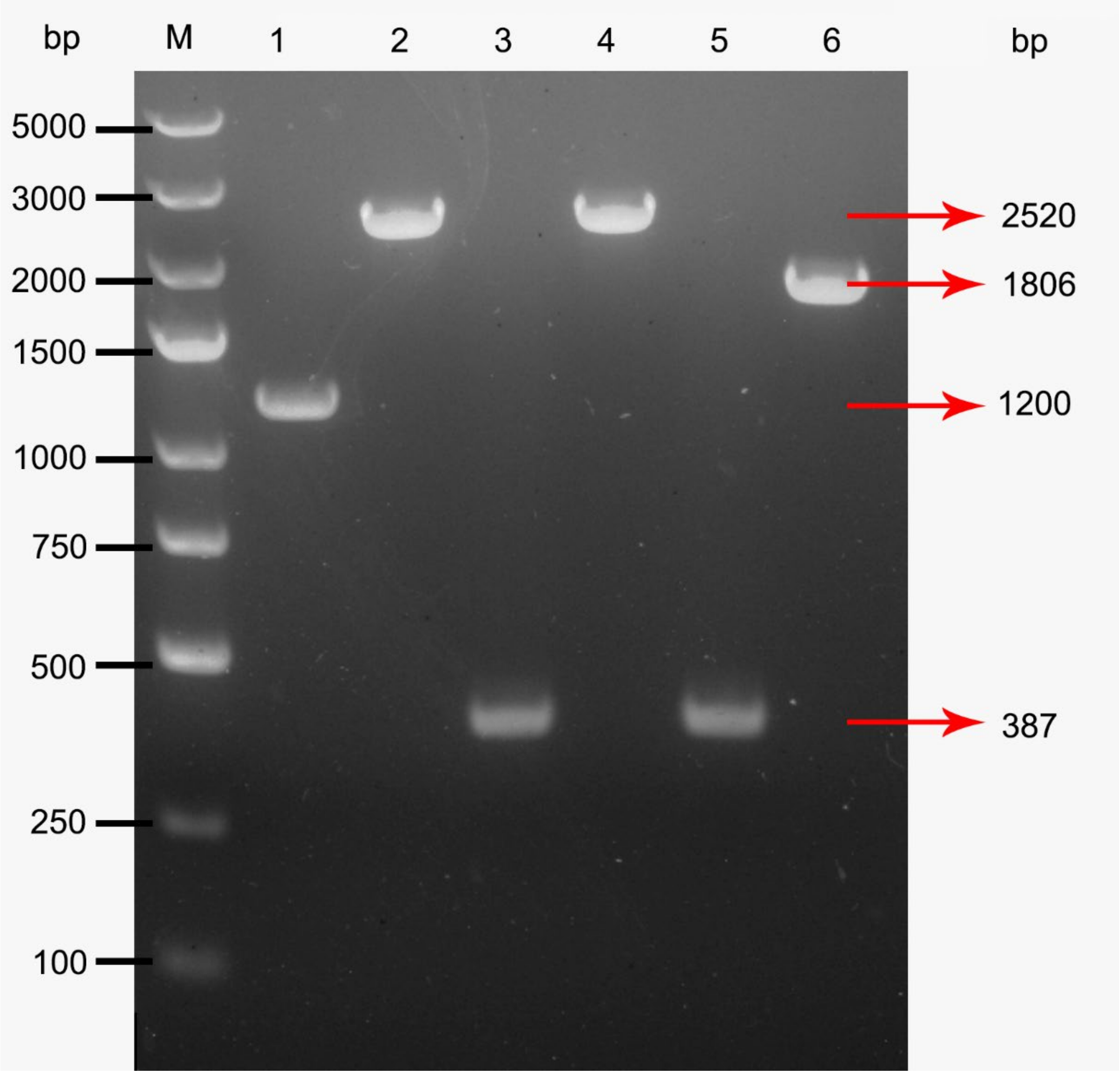

Figure S2. Identification of the *cwlE* deletion mutant strains Bt-59 (Δ*cwlE*) and Bt-59 (Δ*cwlE-sigK*) by PCR. The PCR products in lanes 1, 3, and 5 were amplified using the genomic DNA of Bt-59, Bt-59 (Δ*cwlE*), and Bt-59 (Δ*cwlE-sigK*) as templates, and cwlE-F/cwlE-R as primers, respectively. The PCR products in lanes 2, 4, and 6 were amplified using the genomic DNA of Bt-59, Bt-59 (Δ*cwlE*), and Bt-59 (Δ*cwlE-sigK*) as templates, and sigK-F/sigK-R as primers, respectively.

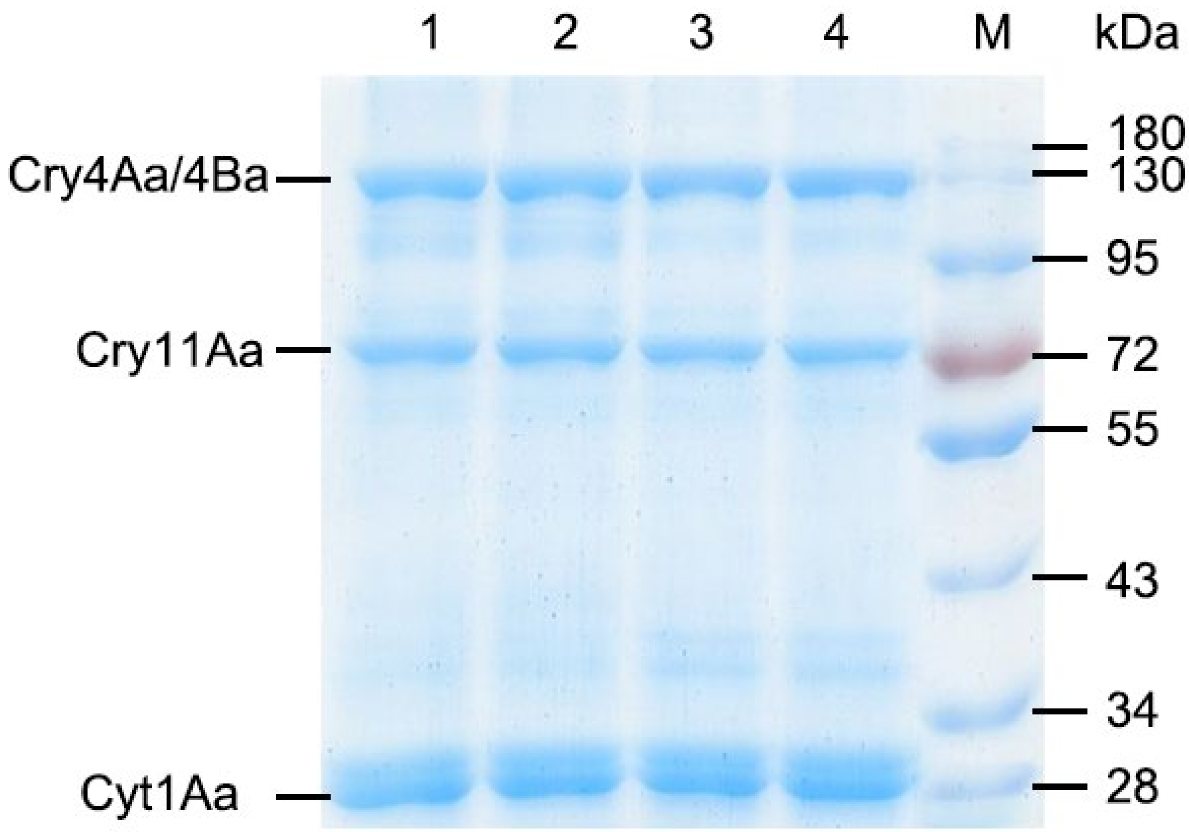

Figure S3. Cry proteins production of Bt-59, Bt-59 (Δ*cwlE*), Bt-59 (Δ*sigK*), and Bt-59 (Δ*cwlE-sigK*) were determined by SDS-PAGE. M, protein molecular size marker. The crystal protein production of Bt-59, Bt-59 (Δ*cwlE*), Bt-59 (Δ*sigK*), and Bt-59 (Δ*cwlE-sigK*) were showed in lanes 1, 2, 3, and 4 represent, respectively.

Table S1 Primers used for recombinant plasmids construction, strains identification, and qPCR.

Primer	Sequence (5'-3')
cwlE-1	CAGATCTATCGATGCATGCCATGGTACCCGAATCCATCTGTCCCGCTTAAACTG
cwlE-2	CATGGGAATATTCGGTGTGGGAAATTACATATCCATCGCTCCTCTTCTCTTAG
cwlE-3	CTAAGAGAAGAGGAGCGATGGATATGTAATTTCCCACACCGAATATTCCCATG
	CGTCGACGCGTCTGCAGAAGCTTCTAGAATTCACATACGCAGGGAACATTTACA
cwlE-4	TAATG
HFcwlE-1	GCTATGACCATGATTACGCCAAGCTTTCCATCTGTCCCGCTTAAACTG
HFcwlE-2	GTTGTAAAACGACGGCCAGTGAATTCACGCAGGGAACATTTACATAATG
cwlE-F	AGACAATATCGCTTAATGCGTGAA
cwlE-R	CTAGCTGCCTGTAGCCAGACC
sigK-F	TTCCGGTTCTTGCTGTAAATTAAG
sigK-R	CTATTAACAAACATGGCACCTAC
pET-cwlE-F	CAGCAAATGGGTCGCGGATCCGAATTCATGGATATGGTTAAAGTTTGG
pET-cwlE-R	GGTGCTCGAGTGCGGCCGCAAGCTTTTAATTTAATCTTTTTTTCCAAG
PcwlE-F	AACTGCAGCGGATGACTTTCTACAACTAC
PcwlE-R	CGGGATCCCGCTCCTCTTCTCTTAGTACT
RpsU-F	AAGATCGGTTTCTAAAACTGGTACA
RpsU-R	TTTCTTGCCGCTTCAGATTT
gatB-F	AGCTGGTCGTGAAGACCTTG
gatB R	CGGCATAACAGCAGTCATCA
qcwlE-F	GTCTCATTCCATTGTAATAGTGGTG
qcwlE-R	TCGATTACGAAGTCCATGTTTAG
qsigK-F	TTGATTGAACATAATTTACGGCTTGT
qsigK-R	GCTTTGTTCCCTTTCCTGCA
qcry4Aa-F	CAGTGCCTATACTATTGTAGTTGGG
qcry4Aa-R	ATTGGTCTTGGGCTGGAAAA
qcry4Ba-F	ACGCTTATGGATTGTGAGGA
qcry4Ba-R	CCTTCGGTTTCGCCTATCTC
qcry11Aa-F	AGGATGGATAGGAAACGGAA
qcry11Aa-R	AAATAGGTCTGCTGGTGCTG
qcyt1Aa-F	CAACAGCAAGGGTTATTACATTA
q cyt1Aa-R	AAAGCGTAGGGCATCACCAA
